# Clinical, Radiographic and Microbiological Evaluation of High Level Laser Therapy, a New Photodynamic Therapy Protocol, in Peri-Implantitis Treatment; a Pilot Experience

**DOI:** 10.1155/2016/6321906

**Published:** 2016-06-09

**Authors:** Gianluigi Caccianiga, Gerard Rey, Marco Baldoni, Alessio Paiusco

**Affiliations:** ^1^Department of Surgery and Translational Medicine, University of Milano-Bicocca, Milan, Italy; ^2^University of Paris Diderot, Paris, France

## Abstract

*Aim*. Endosseous implants are widely used to replace missing teeth but mucositis and peri-implantitis are the most frequent long-term complications related with dental implants. Removing all bacterial deposits on contaminated implant surface is very difficult due to implant surface morphology. The aim of this study was to evaluate the bactericidal potential of photodynamic therapy by using a new high level laser irradiation protocol associated with hydrogen peroxide in peri-implantitis.* Materials and Methods*. 10 patients affected by peri-implantitis were selected for this study. Medical history, photographic documentation, periodontal examination, and periapical radiographs were collected at baseline and 6 months after surgery. Microbiological analysis was performed with PCR Real Time. Each patient underwent nonsurgical periodontal therapy and surgery combined with photodynamic therapy according to High Level Laser Therapy protocol.* Results*. All peri-implant pockets were treated successfully, without having any complication and not showing significant differences in results. All clinical parameters showed an improvement, with a decrease of Plaque Index (average decrease of 65%, range 23–86%), bleeding on probing (average decrease of 66%, range 26–80%), and probing depth (average decrease of 1,6 mm, range 0,46–2,6 mm). Periapical radiographs at 6 months after surgery showed a complete radiographic filling of peri-implant defect around implants treated. Results showed a decrease of total bacterial count and of all bacterial species, except for* Eikenella corrodens*, 6 months after surgery.* Conclusion*. Photodynamic therapy using HLLT appears to be a good adjunct to surgical treatment of peri-implantitis.

## 1. Introduction

Endosseous implants have become widely accepted treatment options for the replacement of missing teeth; the increasing use of implants has led clinicians to observe a higher frequency of peri-implant pathologies [1]. Mucositis and peri-implantitis, defined as inflammatory processes in the tissues surrounding an implant, are the most frequent long-term complications related with dental implants [2].

Peri-implantitis is a bacterially induced inflammatory reaction that results in loss of supporting bone around an implant in function, which may eventually lead to loss of the implant fixture (implant failure). Peri-implant mucositis is a reversible inflammatory process in the soft tissues surrounding a functioning implant, while peri-implantitis is an inflammation of peri-implant tissues accompanied with changes in the level of crestal bone and with the presence of bleeding on probing and/or suppuration, with or without concomitant deepening of peri-implant pockets [3, 4].

A recent study, investigating 1,497 participants and 6,283 implants, estimated for the frequency of peri-implant mucositis included 63.4% of participants and 30.7% of implants, and those of peri-implantitis were 18.8% of participants and 9.6% of implants [5].

The presence of microorganisms is fundamental for the development of peri-implant disease [6]. Within weeks after the installation of titanium implants, subgingival microflora associated with periodontitis is established. Bacterial colonization and maturation of biofilms depend on a favourable ecological environment and lead to shifts in the composition and behaviour of the endogenous microbiota that may become intolerable for host tissues [7, 8]. A recent study investigated the microbial signatures of the peri-implant microbiome in health and disease using 16S pyrosequencing [9]. Peri-implant biofilms demonstrated significantly lower diversity than subgingival biofilms in both health and disease; however, several species, including previously unsuspected and unknown organisms, were unique to this niche. The peri-implant microbiome differs significantly from the periodontal community in both health and disease. Peri-implantitis is a microbially heterogeneous infection with predominantly Gram-negative species and is less complex than periodontitis.

Therapies currently recommended for the treatment of peri-implantitis are primarily based on scientific evidence resulting from periodontal disease treatment [10]. Biofilm removal from implant surfaces is the primary goal in the treatment of peri-implant disease [11, 12].

Therapies such as antibiotics, antiseptics, and laser treatments have been proposed as additional therapeutic options in nonsurgical treatment of peri-implantitis and mucositis [13]. Also different surgical procedures, sometimes associated with laser irradiation, have been employed to obtain healing and/or regeneration of defects in patients with peri-implantitis [14].

Cumulative Interceptive Supportive Therapy (CIST), proposed by Lang and Lindhe [15], is a cumulative protocol including four subsequent therapeutic phases, which increase antimicrobial potential depending on lesion extent and severity.

Surgical therapy is first-choice treatment for peri-implantitis because of lesion and compromised implant surface complexity [16].

Surgery main goal is to create access for debridement and decontamination of contaminated implant surface. Biofilm and calcified deposits must be removed in order to allow healing and reduce the risk for disease future progression [17, 18].

Mechanical instrumentation should be followed by chemical decontamination of the implant surface. Different solutions have been used, including citric acid, chloramines, tetracycline, chlorhexidine, hydrogen peroxide, and sodium chloride. No method was superior to the other [19].

Studies from literature show that regenerative surgical therapy of peri-implantitis presents some controversial issues, such as the real possibility to obtain decontamination of implant surface, regeneration of lost bone tissue, and reosteointegration of implant surface [20, 21].

Lasers were introduced into medicine in 1964 [22] and are now successfully widely employed in dentistry for treatment of different pathologies. Recently, an increasing number of studies evaluating the efficacy of photodynamic therapy for periodontal diseases treatment have been published [23, 24].

Photodynamic therapy (PDT) can be defined as eradication of target cells by reactive oxygen species produced by means of a photosensitizing compound and light of an appropriate wavelength. It could provide an alternative for targeting microbes directly at the site of infection, thus overcoming the problems associated with antimicrobials. Photodynamic action describes a process in which light, after being absorbed by dyes, sensitizes organisms for visible light induced cell damage [25].

At the beginning of the last century, researchers found that microbes became susceptible to visible light mixed with a photosensitizing compound. Raab et al. first showed the killing of protozoa* Paramecium caudatum* in the presence of acridine orange when irradiated with light in the visible range of spectrum. This combination of two nontoxic elements, dye and light, in an oxygenated environment induces damage and total destruction of microorganisms. In 1904, Von Tappeiner and Jodlbauer coined the term photodynamic to describe oxygen-dependent chemical reactions induced by photosensitization which could inactivate bacteria [26].

PDT involves three components: photosensitizer, light, and oxygen. When a photosensitizer is irradiated with light of specific wavelength it undergoes a transition from a low-energy ground state to an excited singlet state. Subsequently, the photosensitizer may decay back to its ground state, with emission of fluorescence, or may undergo a transition to a higher-energy triplet state. The triplet state can react with endogenous oxygen to produce singlet oxygen and other radical species, causing a rapid and selective destruction of the target tissue.

PDT produces cytotoxic effects on subcellular organelles and molecules. Its effects are targeted on mitochondria, lysosomes, cell membranes, and nuclei of tumor cells. Photosensitizer induces apoptosis in mitochondria and necrosis in lysosomes and cell membranes.

The aim of this study was to evaluate the bactericidal potential of photodynamic therapy by using a new high level laser irradiation protocol associated with hydrogen peroxide in peri-implantitis.

## 2. Materials and Methods

### 2.1. Study Population

We selected 10 patients for this study affected by peri-implantitis.

Patient selection was guided by precise inclusion and exclusion criteria:

- Age between 35 and 70 years old.
- Presence of peri-implantitis which did not undergo surgical treatment in the last 12 months. At least peri-implant pockets >4 mm with bleeding on probing.
- Nonsmoking history.
- Absence of allergies.
- Absence of uncontrolled systemic disease.
- Absence of antibiotic therapy in the last 6 months.
- Absence of pregnancy or lactating.
- Absence of abuse of alcohol or drugs.
- Acceptance of the surgical intervention by signing an informed consensus.

We decide not to impose restriction about the gender of the patients (male or female).

### 2.2. Clinical, Radiographic, and Microbiological Parameters

**Figure 1:**
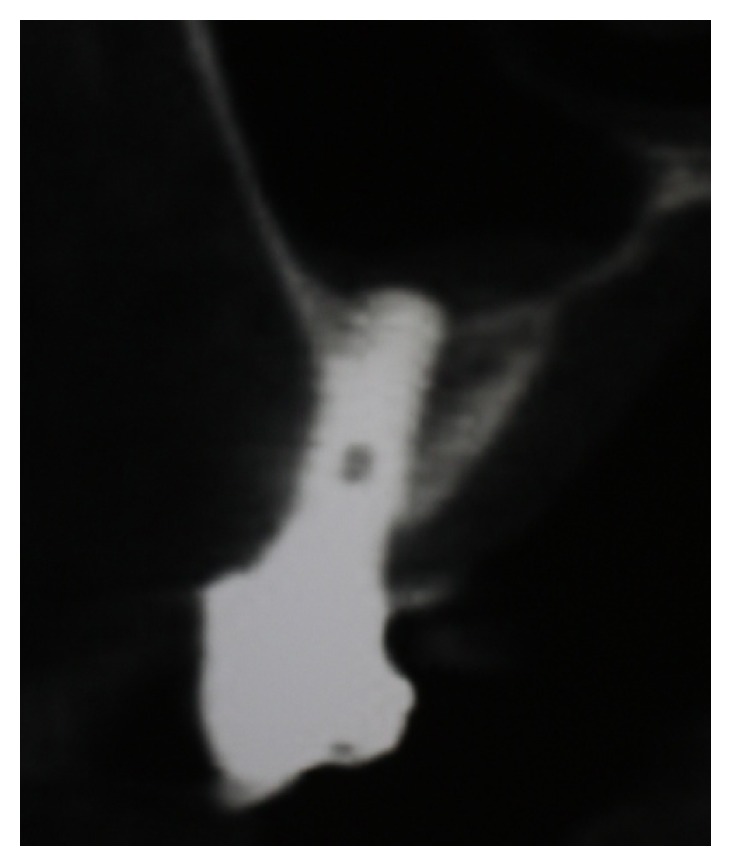
Initial radiograph.

The initial treatment consisted of a medical history, photographic documentation, periodontal examination, and periapical radiographs (Figure 1).

Data were collected at baseline and 6 months after surgery.

For each patient periodontal charting was performed, assessing probing depth, Plaque Index, and bleeding on probing. Microbiological analysis was performed with PCR Real Time, using paper tips to withdraw gingival fluid in peri-implant pockets before and after treatment.

### 2.3. Presurgical Procedures

One week before surgery each patient underwent nonsurgical periodontal therapy combined with photodynamic therapy according to High Level Laser Therapy protocol.

Scaling and root planing of all periodontal and peri-implant pockets was performed using Gracey curettes and ultrasonic instruments combined with Betadine (5 : 1 ratio) irrigation and air powder abrasive device with sodium bicarbonate powder.

### 2.4. High Level Laser Therapy Protocol

Photodynamic therapy was applied using Oxylaser solution (hydrogen peroxide stabilized with glycerophosphoric complex) and high power diode laser with the following parameters:

- Power: 2.5 W.
- Frequency: 10.0 kHz.
- T-on 20 *μ*s, T-off 80 *μ*s.
- Mean power: 0.5 W.
- 60 seconds per site.
- Fiber: 400 microns.

Oxylaser solution was irrigated in each periodontal and peri-implant pocket, that emerging from gingival sulcus was aspirated, and remaining part was left in site for two minutes.

Laser fiber was introduced within the pocket, reaching the bottom and radiating subgingival tissues with a movement back and forth 60 seconds for each single pocket.

### 2.5. Surgical Procedures

**Figure 2:**
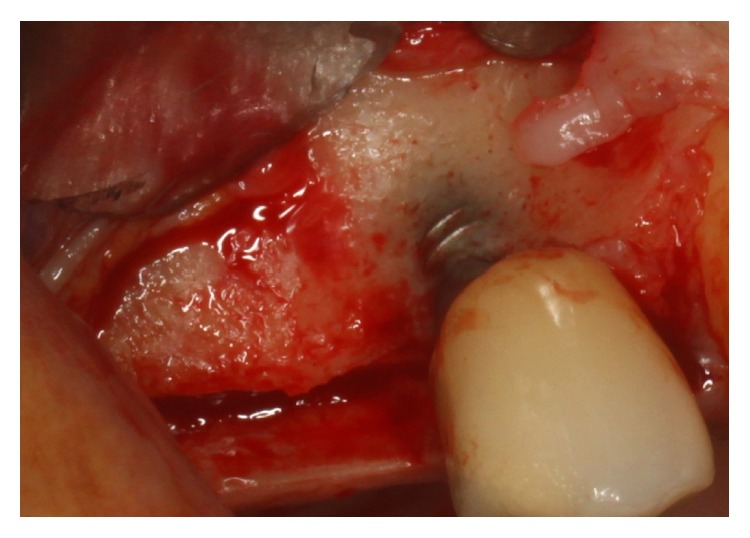
Peri-implant defect.

**Figure 3:**
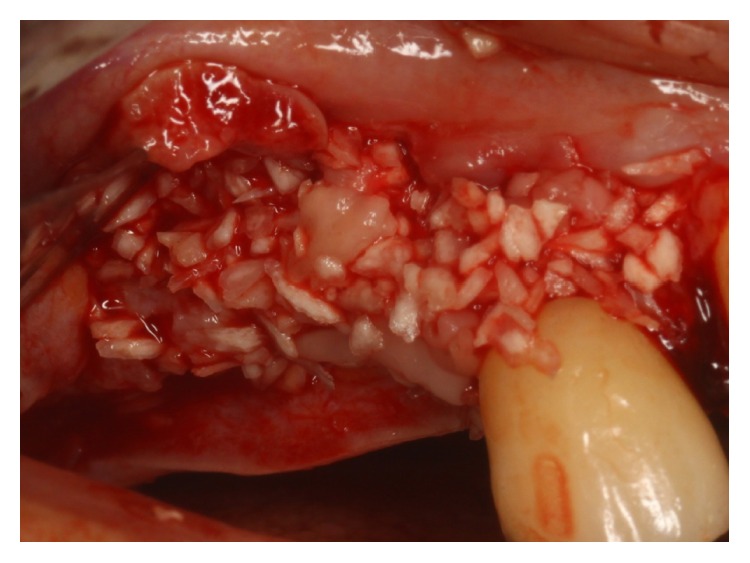
Bone graft after degranulation and HLLT.

**Figure 4:**
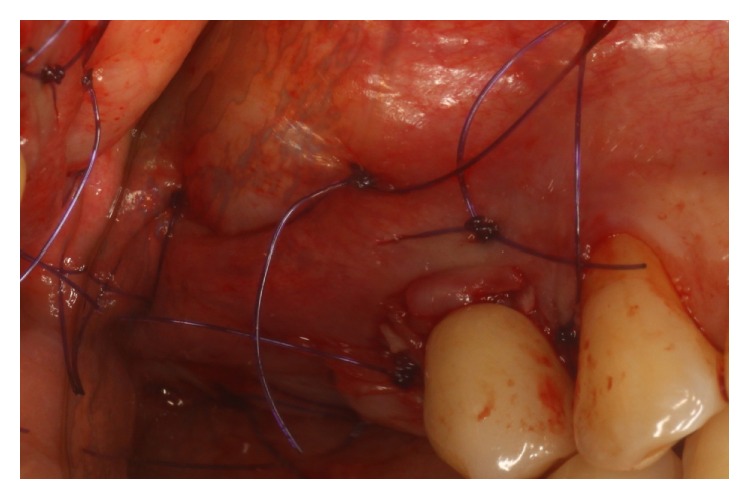
Sutures.

Surgical procedures were performed under local anesthesia. Intrasulcular incisions were performed and a full thickness mucoperiosteal flap was elevated to expose both the labial and palatal aspects of peri-implant defect (Figure 2). Granulation tissue was curetted and removed by using Gracey curettes and ultrasonic instruments combined with Betadine (5 : 1 ratio) irrigation and air powder abrasive device with sodium bicarbonate powder. High level laser irradiation was applied on implant surface 60 seconds for each single pocket and debridement procedures were repeated until complete cleaning of the implant surface. After bone grafting (Figure 3) full thickness buccal and lingual flaps were repositioned and sutured (Figure 4), giving a first internal mattress suture to remove flap tensions.

### 2.6. Follow-Up

**Figure 5:**
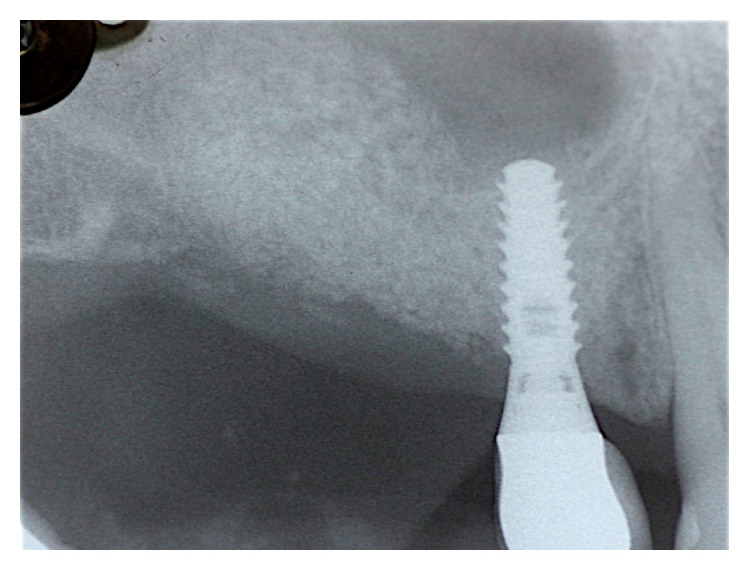
Radiograph 6 months after surgery.

**Figure 6 fig6:**
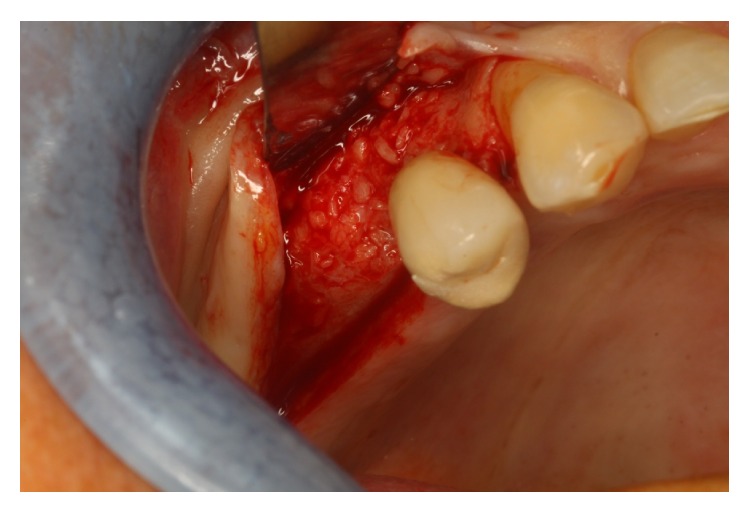
Reentry surgery for implant placement showing new bone formation on implant treated.

**Figure 7 fig7:**
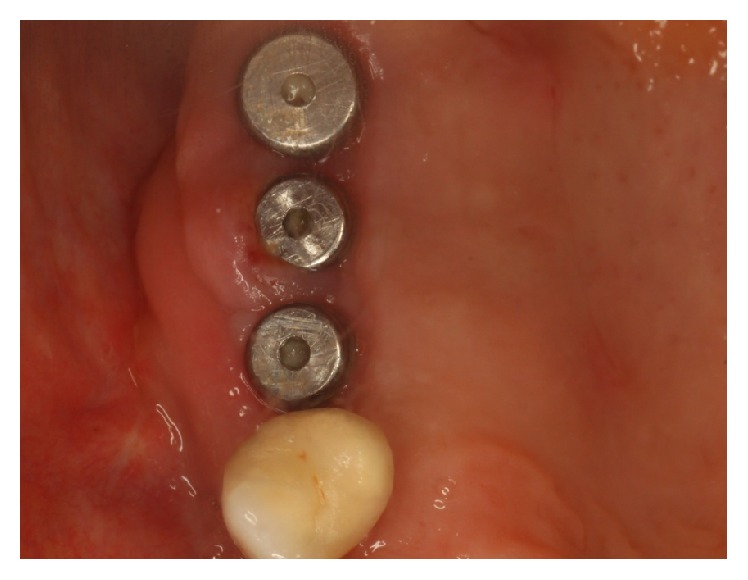
Implant placement in regenerated bone.

**Figure 8:**
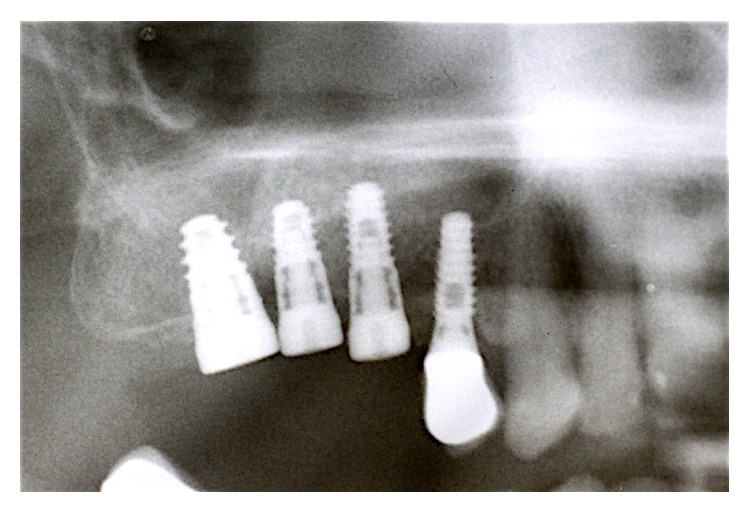
Radiographic evaluation after implant placement.

Sutures were removed 15 days after surgery and High Level Laser Therapy was performed to allow further decontamination. Every 20 days for 3 months patients underwent HLLT. 6 months after surgery clinical, radiographic, and microbiological data were collected (Figures 5 [Fig fig6] [Fig fig7]–8).

## 3. Results

Initially 12 patients were considered for this study, but 2 were excluded due to the following reasons: 1 patient had uncontrolled diabetes mellitus and 1 patient did not follow hygiene instructions.

All 10 patients included in the study (4 males and 6 females; average age 48,6 years; range between 35 and 63 years) agreed to undergo surgery and High Level Laser Therapy.

Implants treated in this study were

- 4 Nobel implants with TiUnite surface,
- 3 Straumann implants with SLA surface (one represented in the case report),
- 1 Straumann implant with SLActive surface,
- 2 Zimmer implants with MTX surface.

All peri-implant pockets were treated successfully, without having any complication and not showing significant differences in results.

**Figure 9:**
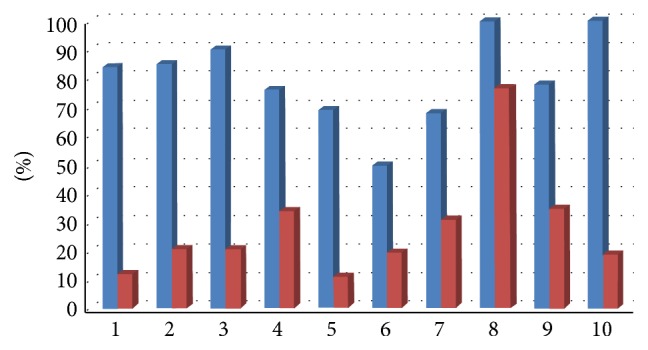
Plaque Index at baseline and 6 months after therapy.

**Figure 10:**
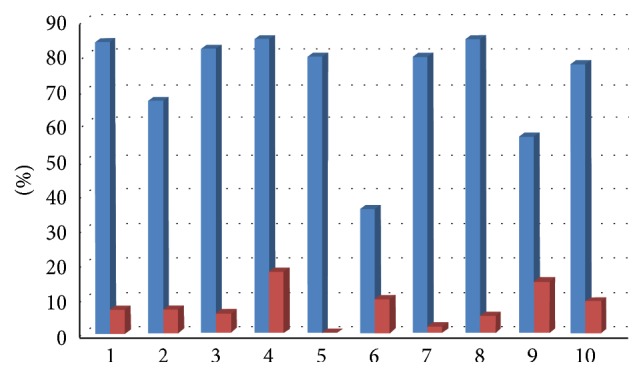
Bleeding on probing at baseline and 6 months after therapy.

**Figure 11:**
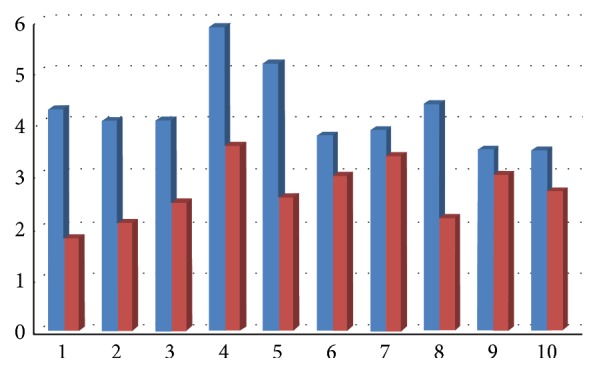
Probing depth at baseline and 6 months after therapy.

All clinical parameters showed an improvement, with a decrease of Plaque Index (average decrease of 65%, range 23–86%, Figure 9), bleeding on probing (average decrease of 66%, range 26–80%, Figure 10), and probing depth (average decrease of 1,6 mm, range 0,46–2,6 mm, Figure 11).

Periapical radiographs at 6 months after surgery showed a complete radiographic filling of peri-implant defect around implants treated.

Microbiological analysis was carried out on different bacterial species, including* Aggregatibacter actinomycetemcomitans* (Aa),* Porphyromonas gingivalis* (Pg),* Tannerella forsythia* (Tf),* Treponema denticola* (Td),* Fusobacterium nucleatum* (Fn),* Campylobacter rectus* (Cr), and* Eikenella corrodens* (Ec) and on total bacterial count.

**Figure 12:**
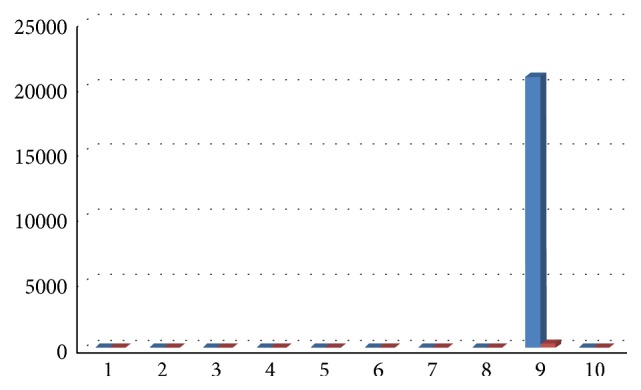
Aa: microbiological analysis at baseline and 6 months after surgery.

**Figure 13:**
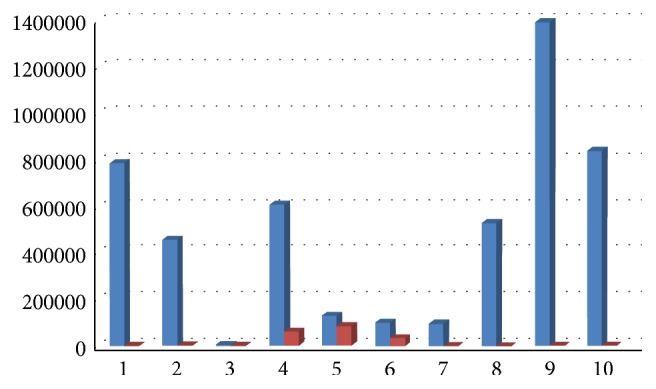
Pg: microbiological analysis at baseline and 6 months after surgery.

**Figure 14:**
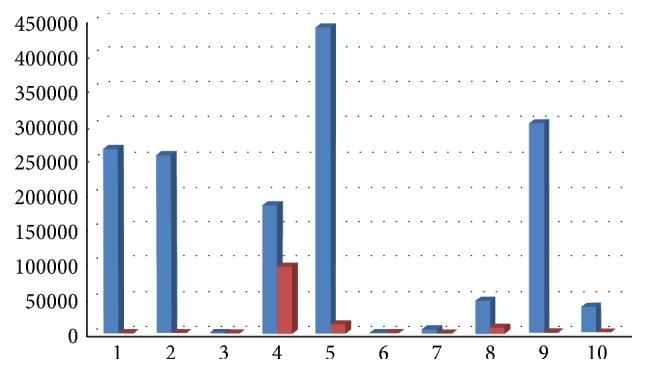
Tf: microbiological analysis at baseline and 6 months after surgery.

**Figure 15:**
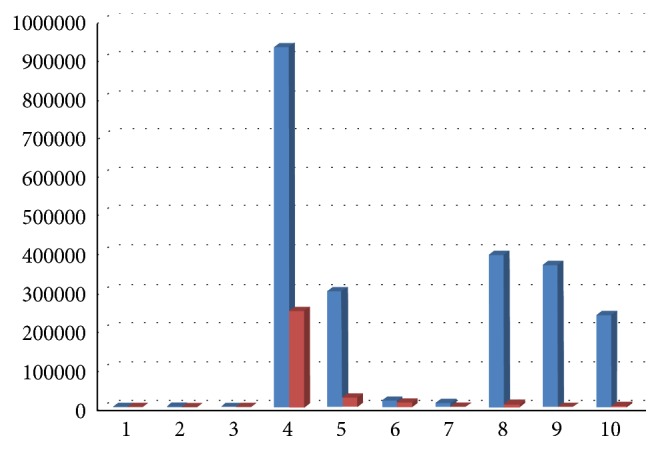
Td: microbiological analysis at baseline and 6 months after surgery.

**Figure 16:**
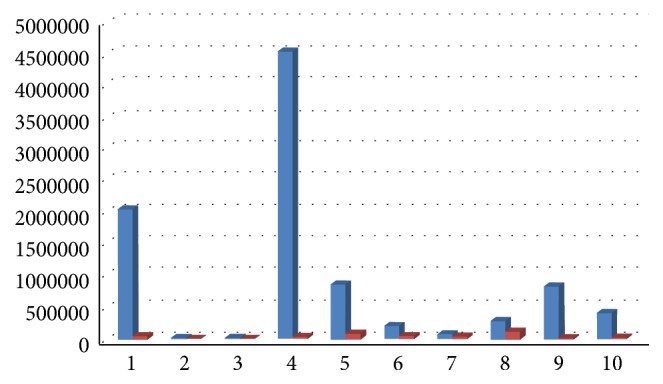
Fn: microbiological analysis at baseline and 6 months after surgery.

**Figure 17:**
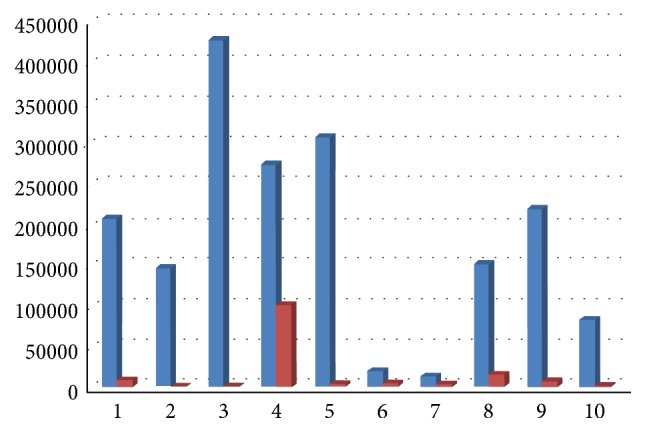
Cr: microbiological analysis at baseline and 6 months after surgery.

**Figure 18:**
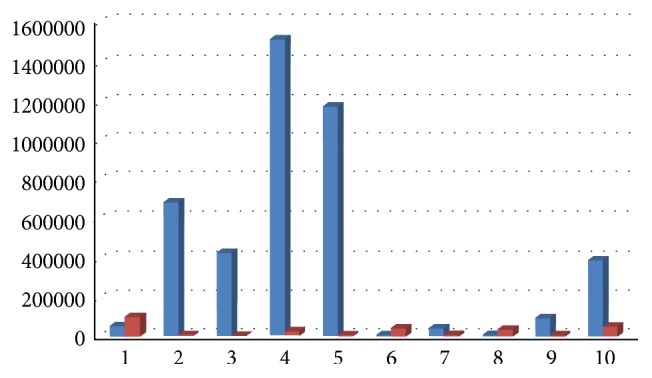
Ec: microbiological analysis at baseline and 6 months after surgery.

**Figure 19:**
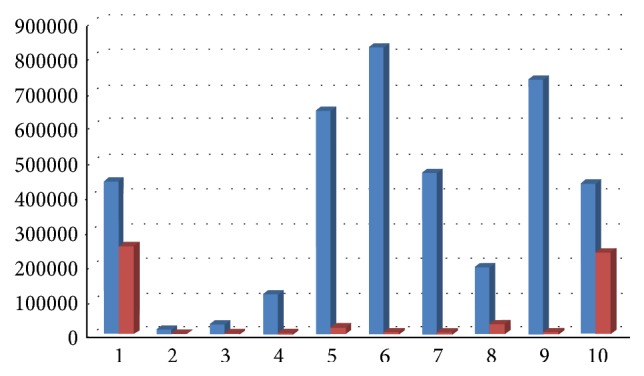
Total microbial count: microbiological analysis at baseline and 6 months after surgery.

Results showed a decrease of total bacterial count and of all bacterial species, except for Ec, 6 months after surgery, with a medium decrease of 98,70% for Aa (Figure 12), 89% for Pg (range 100%–34,55%, Figure 13), 92% for Tf (range 100%–34,55%, Figure 14), 88% for Td (range 100%–34,55%, Figure 15), 85,68% for Fn (range 100%–34,55%, Figure 16), 89,64% for Cr (range 100%–34,55%, Figure 17), and 85,27% for total bacterial count (range 100%–34,55%, Figure 19). Ec showed a medium increase of 38,64% (range 100%–491,07%, Figure 18).

## 4. Discussion

Peri-implant surfaces exposed to peri-implantitis, particularly rough ones, promote plaque accumulation and defect evolution both in the dog [2] and in humans [27] but, if decontaminated, may regain original osteophilic ability.

The prerequisite for obtaining reosteointegration of a rough implant surface exposed by bone loss is deep decontamination of bacterial biofilm.

This can be realized with mechanical instrumentation, antiseptics, pharmacological, or photodynamic devices, considering that the primary aim is the removal of toxins and bacteria without permanence of antiseptics or alteration of implant morphological and osteophilic characteristics.

Mechanical treatment alone is not able to remove all the biofilm due to implant morphology and roughness, so it should be integrated with antiseptic or pharmacological devices.

The use of a simple system as the combination of CHX and saline solution at 0.2% could be sufficient to decontaminate implant surface as shown by Singh [28] in a study on monkeys in which researchers have achieved 39–46% of reosteointegration with this surface treatment through regenerative techniques (autogenous bone + ePTFE).

Even Kolonidis et al. [29] have obtained implant surface reosteointegration in a dog model after treatment with citric acid or H_2_O_2_ or saline solution.

However, complete decontamination of a rough implant surface is very difficult to achieve.

A recent study attempted to assess the cleaning potential of three different instrumentation methods commonly used for implant surface decontamination in vitro, using a bone defect-simulating model. None of the cleaning procedures performed, including Gracey curette, an ultrasonic device, and an air powder abrasive device with glycine powder, was able to perfectly clean implant surface [30].

A treatment option to achieve this fundamental goal could be represented by photodynamic therapy, in particular by High Level Laser Therapy technology.

The HLLT technology is a therapy based on the combination of a penetrating laser with a modified and stabilized H_2_O_2_ solution.

Several in vitro studies showed bactericidal activity of laser irradiation combined with hydrogen peroxide on numerous bacterial species.

A comparative study on the effects of laser alone and combined with H_2_O_2_ showed these results [31–34]:

- Laser used alone produces poor results in the elimination of bacterial species involved in periodontal disease.
- H_2_O_2_ used alone produces little effects in microorganisms elimination.
- Laser combined with hydrogen peroxide shows an antibacterial action much more effective on most of the microorganisms involved in periodontal disease.

Laser energy activates the modified H_2_O_2_ solution, releasing free radicals and singlet oxygen that have antibacterial activity on Gram-positive and Gram-negative periodontal pathogens. The photochemical effect of this photodynamic therapy consists of activation of a photosensitizer (in this case hydrogen peroxide), with a monochromatic beam, as the laser beam characterized by a single wavelength. The interaction between this photosensitizer and the laser produces photochemical reactions in which the energy acceptor is oxygen. The stabilized hydrogen peroxide contains oxygen, and its presence allows the reactions of photoactivation and production of singlet oxygen. The singlet oxygen is an oxygen free radical that determines bacterial cells death (destruction of bacterial membrane, degradation of lysosomal membrane, alteration of mitochondrial function, and denaturation of DNA molecules).

Results showed a decrease of total bacterial count and of all bacterial species, except for* Eikenella corrodens*. Analyzing microbiological results regarding* Ec* we found that 7 patients had a medium decrease of 94,42% (range 85,26%–100%) and only 3 patients had a medium increase of 347,95% (range 73,47%–491,07%). In vitro studies we published in the last years, evaluating the efficacy of this protocol on different bacterial species, suggested that HLLT protocol is able to deplete all bacteria examined. Therefore recolonization of treated peri-implant pockets in these 3 patients by* Eikenella corrodens* is more likely than a persistence in the pocket of this bacterial species. Recolonization could be related to different factors, especially poor oral hygiene (confirmed in these 3 patients).

It is important to understand that this laser works at high power peaks (to kill bacteria), at reduced values of average power (below 0.8 watts), and with a very high frequency. All this is allowed by the fact that this laser works in microseconds and not in milliseconds, greatly increasing the frequency. The strong increase in the frequency (in the study consisting of 20 microseconds to 80 microseconds of T-on and T-off) allows the use of very high peak power (2.5 W) while maintaining an average power below the 0.8 watts, without having any thermal effect.

Summarizing the HLLT it is characterized by

- high peak power (2.5 watts): allowing the destruction of microorganisms (decontaminating effect),
- reduced average power (0.5 watts) and timing of application reduced: reducing high thermal effects that are harmful to the tissues, resulting in only mild thermal effects (increased vasodilation), which increases blood flow to the site of intervention promoting healing and regeneration (increased intake of growth factors, oxygen, inflammatory, and stem cells),
- high frequency (10,000 Hz): important activation and release of singlet oxygen (10,000 times per second) that increase the antibacterial activity,
- maximum depth of penetration: with HLLT the photosensitizer used is oxygen-rich and transparent, increasing laser penetration depth compared to chromophores,
- elimination of silver compounds by H_2_O_2_ and stabilization with glycerol-phosphate that has biostimulating effects.

The proposed protocol does not rely only on photodynamic therapy but combines all the chemical and mechanical actions of the conventional nonsurgical therapy (sonic and curette instrumentation).

Peri-implant treatment relies on different types of action:

- Mechanical action (scaling with sonic instruments and/or curettes).
- Chemical action (sonic irrigation with Betadine, in solution 1/5).
- Mechanical and chemical action of air flow with high abrasive bicarbonate powder.
- Physical action (photodynamic therapy): effective in eliminating even the most aggressive bacteria.

The combination of these three phases during therapy allows a deep disinfection on any implant surface.

In HLLT laser is set so as to avoid significant thermal effects, which does not modify the implant surface. The decontamination is performed with both nonsurgical and surgical protocol, with the combined use of sonic, chemical, physical, and photodynamic devices.

## 5. Conclusions

The majority of analyzed studies show modest beneficial effects of pulsed lasers in comparison to conventional therapies (with manual and/or sonic instrumentation) in the initial treatment of patients with peri-implantitis. Photodynamic therapy using HLLT, supported by a biological rationale and by preliminary results obtained with this study, appears to be a good adjunct to surgical treatment of peri-implantitis; the efficacy of the proposed protocol highlights the need to act on the site as less traumatically as possible but in an effective way in order to improve the bacterial and inflammatory condition.

Reduced periodontal inflammation, with a decrease in probing depth and bleeding on probing, and the massive reduction of bacteria, particularly aggressive pathogens often found in affected sites, are suggestive of the potential effectiveness of this protocol for the treatment of peri-implant disease.
